# Take the Money and Run: Psychopathic Behavior in the Trust Game

**DOI:** 10.3389/fpsyg.2016.01866

**Published:** 2016-11-28

**Authors:** Manuel I. Ibáñez, Gerardo Sabater-Grande, Iván Barreda-Tarrazona, Laura Mezquita, Sandra López-Ovejero, Helena Villa, Pandelis Perakakis, Generós Ortet, Aurora García-Gallego, Nikolaos Georgantzís

**Affiliations:** ^1^Department of Basic and Clinical Psychology, Universitat Jaume ICastelló, Spain; ^2^Centre for Biomedical Research Network on Mental Health, Instituto de Salud Carlos IIIMadrid, Spain; ^3^Laboratory of Experimental Economics and Economics Department, Universitat Jaume ICastellón, Spain; ^4^Centro de Investigación Mente, Cerebro y Comportamiento, Universidad de GranadaGranada, Spain; ^5^School of Agriculture, Policy and Development, University of ReadingReading, UK

**Keywords:** behavioral economics, psychopathy, personality, experiment, trust game, risk attitudes

## Abstract

We study the association among different sources of individual differences such as personality, cognitive ability and risk attitudes with trust and reciprocate behavior in an incentivized experimental binary trust game in a sample of 220 (138 females) undergraduate students. The game involves two players, player 1 (P1) and player 2 (P2). In the first stage, P1 decides whether to trust and let P2 decide, or to secure an egalitarian payoff for both players. If P1 trusts P2, the latter can choose between a symmetric payoff that is double than the secure alternative discarded by P1, and an asymmetric payoff in which P2 earns more than in any other case but makes P1 worse off. Before the main experiment, we obtained participants’ scores for Abstract Reasoning (AR), risk attitudes, basic personality characteristics, and specific traits such as psychopathy and impulsivity. During the main experiment, we measured Heart Rate (HR) and ElectroDermal Activity (EDA) variation to account for emotional arousal caused by the decision and feedback processes. Our main findings indicate that, on one hand, P1 trust behavior associates to *positive emotionality* and, specifically, to the extraversion’s *warmth* facet. In addition, the impulsivity facet of *positive urgency* also favors trust behavior. No relation to trusting behavior was found for either other major personality aspects or risk attitudes. The physiological results show that participants scoring high in psychopathy exhibit increased EDA and reduced evoked HR deceleration at the moment in which they are asked to decide whether or not to trust. Regarding P2, we find that AR ability and mainly low *disagreeable disinhibition* favor reciprocal behavior. Specifically, lack of reciprocity significantly relates with a psychopathic, highly disinhibited and impulsive personality. Thus, the present study suggests that personality characteristics would play a significant role in different behaviors underlying cooperation, with extraversion/*positive emotionality* being more relevant for initiating cooperation, and low *disagreeable disinhibition* for maintaining it.

## Introduction

Cooperation between strangers is an essential characteristic of human societies that differentiates us from other animal species (Fehr and Fischbacher, 2003). Central processes for understanding such cooperation are trust and reciprocity (Nowak, 2006; Walker and Ostrom, 2009; Balliet and van Lange, 2013). In accordance to the centrality of these behaviors for important social, economic and political outcomes, they have become a relevant topic in classic disciplines, such as anthropology, sociology, evolutionary biology, psychology or economics, and in new emerging interdisciplinary fields, such as neuroeconomics (Loewenstein et al., 2008) and behavioral economics (Kahneman, 2003; Camerer et al., 2011). One of the most powerful tools for the development of these fields has been the use of economic games (Evans and Krueger, 2009; King-Casas and Chiu, 2012; Sharp, 2012). Economic games are multiplayer decision-making tasks originally developed within mathematical theory to analyze strategic decision-making among economic agents. Later, they have been extensively used as well-controlled, flexible, and replicable behavioral paradigms to model social interactions such us cooperation, trust, altruism, reciprocity, or retaliation, making them ideal for bridging the gap between theory and naturalistic data (Zhao and Smillie, 2015).

One experimental economic game frequently used for the study of cooperative behavior is the Trust Game^1^ (TG), originally developed by Berg et al. (1995) to measure trust, and to show the importance of positive reciprocity in cooperation. Positive reciprocity is defined as the costly behavior of a second mover (trustee) that reward a kind behavior of the first mover (trustor) (Falk and Fischbacher, 2006), whereas trust in this game would be defined as a voluntary transfer of own money to another subject, with future reciprocation expected but not guaranteed (Gunnthorsdottir et al., 2002). The amount sent by the trustor is multiplied by some factor and received by the trustee, who in turn chooses to send all, some, or none of the received money back to the sender. Although the mathematically computed subgame perfect equilibrium solution of the TG predicts no transfer and no return, there are two main results systematically found: trustors tend to invest positive amounts and trustees to reciprocate to some extent (Johnson and Mislin, 2011).

Importantly, there are individual differences in these behaviors, i.e., people differ quantitatively in the extent of investment of the trustor and the reciprocation of the trustee. Interestingly, a significant portion of these individual differences are attributed to genetic factors, with heritability estimates ranging from 10 to 32% for trust behavior, and from 17 to 32% for trustworthiness, depending on the sample, Swedish or U.S., and the model, ACE or AE, (Cesarini et al., 2008). Personality is also under relevant genetic influences (Vukasovic and Bratko, 2015), and the potential role of personality at the basis of these behaviors has been widely acknowledged (Borghans et al., 2008; Ferguson et al., 2011; Heckman, 2011; Zhao and Smillie, 2015). Thus, the main objective of the present study is to explain (part of) these individual differences by means of personality characteristics. Our major strength and novelty is that we try to explore this association systematically: we assess personality dimensions of the two more relevant personality models of the last decades, the Big Three and the Big Five and explore the role of more specific personality traits. Specifically, we focus on two aspects that could be relevant for collaborative behaviors, not previously examined in the TG: subclinical psychopathy and impulsivity. Examining the personality domains and traits associated to trust and reciprocity will help explaining relevant basic processes underlying cooperative behavior.

Among the most influential personality models in the last decades, those of Eysenck (1992) and McCrae and Costa (2008) are especially relevant for cooperative behavior. In an attempt to link psychological disorders to normal personality, Eysenck (1992) proposed three basic dimensions or facets: Extraversion (E), Neuroticism (N), and Psychoticism (P). P is conceived as normal personality dimension of vulnerability to antisocial behavior and psychopathy, whereas low P would be characterized by traits as empathy, socialization and cooperativeness (Eysenck, 1992). In the other hand, the most widely used and integrative model of personality nowadays is the Five-Factor Model (FFM) (John et al., 2008). This model encompasses five personality dimensions: E, N, Openness to Experience (O), Agreeableness (A) and Conscientiousness (C) (McCrae and Costa, 2008). These domains include specific facets of A and E, such as trust, altruism, straightforwardness, tender-mindedness or warmth that could be especially relevant in interpersonal relationships and for trust and reciprocity (Evans and Revelle, 2008). Consequently, it would be expected that the personality characteristics more relevant in interpersonal behavior, such us A and E would facilitate cooperative behavior, whereas the opposite, exploiting other people and parasitic behavior, would be predicted by low A, P and psychopathic-like characteristics.

Only a few studies have investigated the role of personality domains and their effects on trust and reciprocity in the TG, and no study has explored the role of theoretically relevant specific traits such as impulsivity or psychopathic-like personality. In relation to broad personality dimensions, we deal first with investment behavior of P1, i.e., trust. Although the results are relatively heterogeneous, they tend to show that those personality domains more related to interpersonal behavior, i.e., E and A, were the more consistent personality correlates of trust. Evans and Revelle (2008) found that A was associated with investing, mediated by the trait of trust. Using a strategic version of the TG, Becker et al. (2012) showed a significant correlation between the amount sent as a first mover and A, O and low C. Similarly, Müller and Schwieren (2012) found that the amount sent by the investor correlates significantly with low C and low N, and also significantly positive with A. Swope et al. (2008) found that E was associated with more sending in the TG. Accordingly, Ben-Ner and Halldorsson (2010) found that E and low C were strong predictors of the amount sent to a partner by investors. Also, Haring et al. (2013), using a humanoid robot as a trustee, found that the more extravert a person was, the higher the amount sent in the TG. It is interesting to note that some of these studies found a certain effect of low C on trust behavior (Ben-Ner and Halldorsson, 2010; Becker et al., 2012), probably reflecting the role of low deliberation and impulsivity in the decision to trust or not to trust (Müller and Schwieren, 2012).

With regard to trustee behavior, studies seem to suggest a moderate and consistent role of A on reciprocity and, conversely, of low agreeableness-related traits on exploitation behavior. Thus, Ben-Ner and Halldorsson (2010) found that the only personality domain associated to the proportion of received money that is actually sent back was A. Also, Becker et al. (2012) obtained that reciprocity was significantly correlated with A and O. Similarly, Thielmann and Hilbig (2015a) have found that Honesty-Humility, a domain strongly related to the A (Gaughan et al., 2012), predicts trustee returns in three experiments on different variations of the TG. Last, Lönnqvist et al. (2012) found that those participants being both high on N and low on A transferred back much less than did other participants when receiving low investments.

Lönnqvist et al. (2012) have highlighted the fact that the joint presence of high N and low A is (together with low C) a combination typical of Borderline Personality Disorder (BPD) patients (Saulsman and Page, 2004; Samuel and Widiger, 2008). Accordingly, it has been shown that persons with BPD present a striking deficit in trust and reciprocation. When compared with the healthy controls, BPD patients tend to: (a) transfer a smaller amount of monetary units in a TG when acting as investor (Unoka et al., 2009); and (b) send lower returns when acting as a trustee (King-Casas et al., 2008). It is important to note that a core characteristic of BPD patients is impulsivity, mainly the urgency facets (Whiteside et al., 2005), supporting the above mentioned idea that disinhibition/impulsivity traits may play a role in the TG decisions.

But probably the personality disorder more strongly associated to non-cooperative behavior is Psychopathy. Psychopathy is characterized by traits such as social manipulation, exploitation, egocentrism, irresponsibility, deceitfulness, superficial charm, lack of remorse and shallow affect (Miller et al., 2001), and a central characteristic from an evolutionary perspective would be the success of psychopaths in exploiting social emotions of trust and cooperativeness (Mealey, 1995). In terms of the Five Factor Model, psychopathic characteristics may be understood as the extreme end of a continuum along normal personality functioning, and would be strongly represented in (low) A and (low) C domains (Miller et al., 2001; Miller and Lynam, 2003; Gaughan et al., 2012), with the interpersonal affective components (primary psychopathy) more closely related to low A, and the impulsivity and social deviance features (secondary psychopathy) more closely related to low C (Miller et al., 2008).

Surprisingly, the role of psychopathic characteristics has not been explored yet in the TG, although, studies in other economic games seem to indicate that psychopaths, both clinical and sub-clinical, have a tendency to behave in a non-cooperative way. Mokros et al. (2008) found that criminal psychopaths, compared with healthy participants, were markedly more prone to competitive behavior, as well as to non-adherence to the principles of fairness, as evidenced by greater accumulated reward and exploitation of partners Prisoner’s Dilemma Game (PDG). Similarly, primary-psychopath participants were both less generous to social partners in a dictator game and more likely to reject ungenerous offers in an ultimatum game (Koenigs et al., 2010). Montañés-Rada et al. (2003) found that patients with Antisocial Personality Disorder, a personality disorder strongly related to psychopathy (Widiger and Mullins-Sweatt, 2009), showed more non-cooperative behavior both in the presence and in the absence of a non-cooperative opponent using various modifications of the PDG played against a simulated opponent.

A similar tendency has been observed in non-clinical individuals scoring high on different psychopathy scales. Rilling et al. (2007) found that dyads of high-psychopathy individuals were more likely to lead to mutual defection (non-cooperation) relative to low-psychopathy dyads. In addition, they found a high correlation between non-cooperative behavior and psychopathy scores among the male participants of their sample. Curry et al. (2011), using simultaneous one-shot discrete, continuous and sequential PDG, found that undergraduates with higher scores in Machiavellian Egocentricity PPI subscale, a marker for psychopathy (Benning et al., 2003), cooperated less in simultaneous PDG and were less likely to initiate or reciprocate cooperation in sequential PD games. Gillespie et al. (2013) examine the effects of primary (selfish, uncaring) and secondary (impulsive, irresponsible) psychopathic personality traits on the responses of undergraduate participants to the in-group and the out-group (defined in terms of affiliation to a UK University) in dictator and ultimatum games. They found significant differences in game proposals to members of the in-group and the out-group, between low and high scoring participants on secondary psychopathic traits. Using a PDG with a computerized opponent, Johnston et al. (2014) found that participants with low levels of psychopathic traits exhibited increased social cooperation in the context of affective feedback, and that poor cooperation was uniquely predicted by high levels of psychopathic traits. Taken together, these findings seem to confirm that non-cooperative social actions are the norm among high-psychopathy individuals in social-dilemma, mainly ultimatum and PDG (King-Casas and Chiu, 2012).

Another source of individual differences that could also contribute to cooperative behavior could be general intelligence. Previous research has reported evidence of a positive correlation between intelligence and self-reported trust (e.g., Sturgis et al., 2010; Hooghe et al., 2012; Carl and Billari, 2014). Regarding trust behavior in economic games, a meta-analysis of 36 studies that used a repeated PDG and school-level average SAT and ACT scores as proxies for the intelligence, showed that students cooperate 5–8% more often for every 100-point increase in the school’s average SAT score (Jones, 2008). Similarly, Burks et al. (2009) using a one-shot sequential PDG in a sample of truck driving students found that subjects with greater intelligence more accurately forecast others’ behavior and differentiate their behavior more strongly, depending on the first-mover’s choice. Additionally, players with higher cognitive abilities reciprocated cooperation in the second round of this PDG significantly more than low intelligent subjects. Specifically, in a series of incentivized trust games, Corgnet et al. (2015) showed that cognitive ability is positively correlated to trust but not with trustworthy behavior. Thus, individuals’ cognitive ability/intelligence has been associated with cooperative play in economic games.

Pro-social behavior may also be related to individuals’ risk attitudes. In fact, Luhmann (1988) and Coleman (1990) describe trust from the viewpoint of standard economics as a subclass of situation involving risk. However, Fehr (2009) states that strong neurobiological as well as behavioral evidence indicates that this view is untenable. Accordingly, behavioral studies have consistently failed in finding any relationship between risk aversion and trust behavior in the investment game (e.g., Bohnet and Zeckhauser, 2004; Bohnet et al., 2008; Houser et al., 2010).

Last, the attentional resources and emotional consequences of decision making in the TG are also interesting to study. For example, the conflict between individual and interpersonal considerations may induce different emotional reactions. Also, the attention of a subject in anticipation of the monetary and emotional consequences associated with decision making in the TG could be the result of interaction between the context and a decision maker’s personality. Lorber (2004) investigates the relations of HR and EDA with psychopathy through a meta-analysis of 95 studies. Low resting and task EDA were positively associated with psychopathy, indicating impaired emotional regulation (Casey et al., 2013). Moreover, EDA reactivity was negatively associated with psychopathy. Contrary to the aforementioned relation between EDA and psychopathy, the latter was not associated with HR. In contrast, the relation between cardiac reactivity and psychopathy is less clear (Lorber, 2004; Casey et al., 2013). Here, we investigate these two physiological variables to probe the level of emotional and attentional engagement during the crucial trust decision.

To sum up, collaborative and altruistic behavior is central in human societies. A sequence of trust and reciprocity is usually assumed to be the small-group paradigm equivalent of a society in which citizens trust each other and deserve to be trusted, thus avoiding wasteful use of. An ideal experimental paradigm to examine these behaviors is the TG. In the discrete form adopted here, TG can be seen as a sequential social dilemma type of situation. If P1, who is the first mover, chooses not to trust P2, an egalitarian outcome emerges. Otherwise, if P1 trusts P2, the latter chooses between an egalitarian outcome, which is Pareto-superior to the one discarded by P1 and an unequal one which is favorable to P2 and unfavorable to P1.

The major strength and novelty of this study is that it systematically explores the association between behavior in the trust game with personality and cognitive abilities. To this end, a broad set of personality domains and specific personality facets are assessed. Specifically, we focused on two personality aspects that could be potentially relevant for collaborative behaviors: impulsivity and psychopathy.

No previous studies have directly explored the relationship between psychopathy and behavior in the TG. Considering the non-cooperative, exploitative and parasitic life-style of psychopaths, it is expected that their tendency to benefit from others’ effort and trust would manifest in no reciprocating behavior. Indeed, the TG would be paradigmatic for assessing exploitative and other predatory-related behaviors closely related to psychopathy traits, since one central issue for exploitation is *exploitability*, that is, the observable signs linked with the likelihood of being victimized (Buss and Duntley, 2008). Accordingly, P2’s decision would represent an ideal context for expression of psychopathy-like behavior because P1 is in total *exploitability* by P2, who can benefit from P1’s trust without receiving any negative consequences.

Conversely, agreeable and extraverted individuals tend to show more pro-social behavior, to cooperate more, to trust in other people, even strangers, and to respond in a positive way in front of kind and altruistic behaviors. Thus, A and E constitute the personality pillars of interpersonal relations, with A covering the quality of social interaction and E favoring the quantity of social interaction. Accordingly, one main hypothesis is that trust in the TG would be mainly associated to E and A, whereas reciprocity would be mainly associated to A. Conversely, psychopathy scores would be mainly associated to non-reciprocity and, in a lesser extent, to lack of trust.

Another underexplored area of personality effects on economic games is impulsivity. Impulsivity is a multifaceted construct of emotional-driven facets (positive and negative urgency), cognitive and behavioral features, (lack of both deliberation and perseverance), and sensitivity to reward (sensation seeking) (Whiteside and Lynam, 2001; Cyders et al., 2007). Because of lack of precedents, our hypotheses are general and speculative. In view of the reviewed literature, we hypothesize that impulsivity facets link to positive reinforcement would favor the more rewarding options in the TG, that is, to trust for P1, and to no-reciprocate for P2. Last, we hypothesize a positively relation of trust with cognitive ability and no association with risk-aversion.

## Materials and Methods

### Participants and Procedure

The experiment was run on two different dates. On the first date, 220 (138 females) undergraduate participants were recruited at the Individual Differences and Psychopathology (IDAP) Lab of the Universitat Jaume I. They signed a consent form for the entire experiment which they were informed that would take place on two dates and in two different labs. Then, they were asked to answer different socio-demographic and personality questionnaires.

**FIGURE 1:**
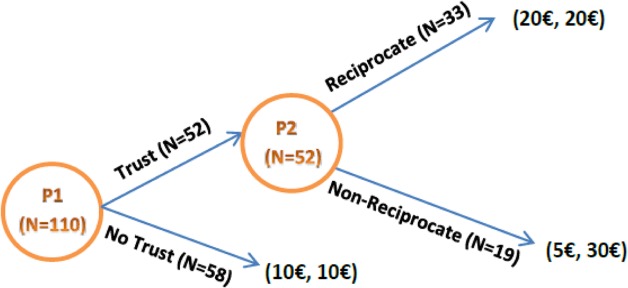
**Extensive form of the TG with real monetary payoffs and numbers of subjects per player type and decision**.

On a second date, the same subjects were invited to the Laboratorio de Economía Experimental (LEE) of the same university to play a TG with real monetary incentives.^2^ We divided the sample in P1 or Trustor (*N* = 110, 71 females) and P2 or Trustee (*N* = 110, 67 females) players (see **Figure 1**). This part of the experiment was carried out in 28 sessions of eight subjects each (forming four random and anonymous pairs per session), using specific software prepared in Java by the IT team at the LEE^3^. The size of groups was dictated by the equipment available in the LEE for measuring Skin Conductance Responses (SCR) and HR variations. Continuous EDA and electrocardiographic (ECG) data were recorded during the entire experimental session using a BIOPAC MP150 system and four TEL100C telemetry modules (BIOPAC systems, Inc.). For EDA acquisition, two Ag/AgCl electrodes filled with isotonic gel were placed on each subject’s distal phalanges of the middle and the index fingers of the non-dominant hand. The skin conductance signal was sampled at 125 Hz and low-pass filtered offline at 0.5 Hz using a Butterworth digital filter. SCR were automatically detected and their amplitudes were quantified using a custom version of the Matlab EDA toolbox.^4^ False SCRs were removed after visual inspection of the entire signal. SCRs were associated to a specific decision if their onset appeared at least 1.0 s after subjects were informed about their possible choices and before the moment of the decision. Only responses above 0.02 microSiemens (μS) were considered as valid.

For ECG acquisition two FLAT active electrodes (Ag/AgCl) were arranged at a modified lead I configuration (i.e., right and left wrists). The ECG signal was sampled at 1000 Hz and filtered offline using a band-pass 0.5 – 50 Hz filter. R-wave detection and artifact correction were performed with the ECGLab Matlab software (Carvalho et al., 2002). We used the KARDIA Matlab software (Perakakis et al., 2010) and custom Matlab scripts (Matlab 2013a, Mathworks Inc.) to analyze the heart period signal during the experimental session. To assess the Phasic Cardiac Responses (PCRs) to a single decision moment, we first calculated the weighted average heart period for a time window of 2 s following the presentation of the decision screen, using the fractional counting procedure described in Dinh et al. (1999). We subsequently subtracted the weighted average heart period calculated for a window 0.5 s before cue onset, in order to express heart period changes as differential values from baseline activity.

### Measures

#### Personality Measures

We used two broad personality models that include impulsivity and psychopathic-related dimensions, i.e., Eysenck’s three factor model and McCrae and Costa’s Five Factor Model, and a more specific test of both impulsivity and psychopathic traits. Importantly, these traits have been closely related to the aforementioned broad personality models (Whiteside and Lynam, 2001; Miller et al., 2008).

The Spanish NEO-PI-R (Costa and McCrae, 1999) is a 240-item self-report measure for quantifying 30 specific traits or facets that define the five personality factors or domains: N, E, O, A, and C. Items are responded to on 5-point Likert scales ranging from 0 (strongly disagree) to 4 (strongly agree). The specific facets for A were: Trust, Straightforwardness, Altruism, Compliance, Modesty and Tendermindedness. For C: Competence, Order, Dutifulness, Achievement striving, Self-discipline and Deliberation. For E: Warmth, Gregariousness, Assertiveness, Activity, Excitement seeking and Positive emotion. For N: Anxiety, Hostility, Depression, Self-Consciousness, Impulsiveness and Vulnerability. Last, for O: Fantasy, Esthetics, Feelings, Actions, Ideas and Values.

The Spanish Short version of the Eysenck Personality Questionnaire-Revised (EPQ-RS; Ortet et al., 2001) assesses Eysenck’s broad dimensions of P, E, and N. Each scale consists of 12 items and the response alternatives are yes/no.

The Spanish version of the Levenson’s Self-Reported Psychopathy Scale (LSRP, Lynam et al., 1999) is a 26-item four-point scale that ranges from 1 (strongly disagree) to 4 (strongly agree). It include two related scales: the LSRP Primary or Factor 1 scale is associated to an antagonistic interpersonal style characteristic of psychopaths (i.e., low A, grandiosity, selfishness, callousness, manipulativeness), whereas LSRP Secondary or Factor 2 scale is more strongly related to disinhibition and negative emotionality (i.e., anger-hostility, urgency, lack of persistence and rashness; Miller et al., 2008; Lynam et al., 2011).

The UPPS-P Impulsive Behavior Scale (Verdejo-García et al., 2010) is a multidimensional inventory that assesses 5 personality pathways contributing to impulsive behavior: negative urgency, positive urgency, lack of perseverance, lack of premeditation, and sensation seeking. The scale is composed of 59 items with a four-point scale that ranges from 1 (strongly agree) to 4 (strongly disagree).

The AR scale of the Differential Aptitude Test (DAT-5, Bennett et al., 2005). This scale consists in a non-verbal AR test. Each item includes four abstract figures following a given rule, and the participant must choose one of five possible alternatives. The score is the total number of correct responses. One advantage of this test is that it is quite fast to implement: it is comprised of 40 multiple-choice items and has a 20 min time limit. AR would be considered a marker of fluid intelligence (Colom et al., 2007), the component of intelligence most related to general intelligence or *g* factor (McGrew, 2009).

#### Risk Attitude Elicitation

We use two different incentive compatible elicitation procedures: the widely used method by Holt and Laury (2002) (Risk aversion HL) and the Sabater-Grande and Georgantzis (2002) lottery-panels (SGG).^5^

Following the HL procedure, subjects are presented with a list of 10 pairwise choices between a safe (S) and a risky (R) lottery, each one of which involves a good and a bad outcome. Then, the difference between the good and the bad outcome in S is smaller than that in R. The list of lottery pairs is created by varying the probability of occurrence of the good outcome from *p* = 0.1 to *p* = 1 in steps of 0.1. A subject’s risk aversion is an increasing function of the number of choices in which he or she has chosen the safe option. Given the monotonicity implied by the design, the actual switching point from S to R is used as the measure of a subject’s risk aversion.

In the lottery panel test, SGG, subjects are faced with eight sub-tasks called panels 1, 2, 3… 8. Panels 1-4 involve only gains, while 5–8 involve mixed gambles. Each panel corresponds to a lottery defined as the probability p of winning a prize X€, else nothing in panels 1–4 (else a fixed loss of 1€ in panels 5–8). In all panels, the winning probability is varied from *p* = 0.1 to *p* = 1 in steps of 0.1. Prizes are designed so that, within a panel, the expected value of lotteries linearly increase in the probability of not winning by a constant t over a fixed gain of 1€ in panels 1–4 and 0€ in panels 5–8. Then, t represents an incentive for subjects to choose riskier choices. This parameter is increased from panel 1 to 4 and from 5 to 8. Thus, intuitively, a subject should be expected to make riskier choices when moving from panel 1 to 4 and from 5 to 8.^6^

In order to estimate the participants’ score in SGG risk attitudes, an exploratory factor analysis with principal axes factor analysis and varimax rotation was performed. According to eigenvalue and parallel analysis, two factors emerged: Factor 1 (Risk aversion F1), comprising Panels 5–8 (with factor loadings from 0.70 to 0.87); and a relatively independent (Factor correlation = 0.20) Factor 2 (Risk aversion F2) comprising Panels 1–4 (with factor loadings from 0.73 to 0.83). These two factors explained 65.5% of the variance.

#### Trust Game

The TG has been implemented in the lab in different versions: framed as a continuous investment game (Costa-Gomes et al., 2014), discrete with multiple choices (Berg et al., 1995) or discrete binary (Gambetta, 1988). Our experimental design is based on a discrete version of the game with binary choices and no particular framing. This strategy aims at reducing the space of investment options in order to facilitate the detection of the cognitive and emotional spectra activated by concentrating the observations on just two possible actions. This has led to more clear-cut data analysis, especially regarding the stimuli homogeneity for emotional arousal studied through the physiological part of our design. In this context, half of the participants acted as P1 players (trustors, *N* = 110), whereas the rest acted as P2 players (trustees).^7^ Instructions to the subjects never mentioned trust, investment or reciprocity, in order to avoid undesirable experimenter demand effects. **Figure 1** presents the payoffs implemented in the game and the number of subjects who chose each strategy.

If the P1 player decides not to trust, both players earn with certainty an amount of 10€ each. But if the P1 player trusts P2, the latter will have to choose whether to reciprocate, raising each players’ earnings to 20€, or to behave individualistically, raising own payoffs to 30€ and letting the trusting player down (5€). Pairs were randomly formed and the game was played once in its genuine sequential form. Each P1 players made the decision whether to trust or not before P2 made the second stage decision, provided that P1 had decided to trust in the first place. As shown in **Figure 1**, 52 (35 females) out of 110 P1 subjects decided to trust. From the 52 active P2 players, 33 (22 females) reciprocated and 19 (11 females) exploited P1’s trust toward them.

### Data Analyses

We conducted the descriptive analyses and calculated correlations among all variables. In order to integrate the highly inter-correlated personality measures and to identify the basic personality domains underlying them, an Exploratory Factor Analysis with the assessed personality dimensions from different bio-dispositional models (NEO-PI-R and EPQ-RS), the measure of psychopathy (LSRP), and the measure of specific facets of impulsivity (UPPS-P) was performed.^8^ We used principal axis factor analysis with varimax rotation. A parallel analysis with the Monte Carlo PA program was carried out to select the number of retained factors. The regression scores for each factor were kept as variables in the database and used later in the regression analysis.

In order to study the relationship among personality, cognitive ability and risk aversion variables on TG behaviors, mean comparison and regression analysis were performed. Thus, *t*-tests were calculated in order to determine whether the differences in personality and intelligence scores between trust vs. no trust groups, and reciprocate vs. no reciprocate groups were statistically significant. In order to examine the role of personality traits on the dichotomous choices in the TG, a Binary Logistic Regression analysis was performed. In a first step, we controlled for potentially confounding variables as age and gender; next, we included the scores on the AR scale of DAT; last, we included factor scores of personality traits. Factor scores were used instead of the 15 direct scores in order to capture the basic personality domains underlying the highly inter-correlated personality scales.^9^ All analyses were performed with the SPSS statistic package, version 21.

## Results

### Descriptive Statistics

In **Table 1** we present descriptive statistics (median and standard deviation) of the explanatory variables included in our study. As usual, women presented higher scores in N, A, and lower scores in psychopathy, P, and several facets of impulsivity (Costa and McCrae, 1999; Ortet et al., 2001; Verdejo-García et al., 2010). In our sample, women also presented lower scores in E and AR. Last, and following Croson and Gneezy (2009) meta-analysis we find that women are in general more risk averse than men in lottery experiments.

**Table 1:** Means, standard deviations and test of differences between men and women (*t*-test for personality variables and MW test for SGG and HL scores on risk attitudes) of the variables included in the study.

	Total (*N* = 222)		Men (*M* = 84)		Women (*F* = 138)		
	*M*	*SD*	*M*	*SD*	*M*	*SD*	Mean comparison
LSRP primary	15.41	6.52	18.49	6.73	13.54	5.64	-5.891ˆ**
LSRP secondary	9.41	3.64	9.51	3.64	9.36	3.65	-0.311
Neuroticism-NEO	92.92	23.40	85.69	24.47	97.33	21.65	3.695ˆ**
Extraversion-NEO	116.80	19.71	120.50	18.86	114.54	19.94	-2.203ˆ*
Openness-NEO	116.59	18.38	114.76	19.70	117.70	17.50	1.157
Agreeableness-NEO	116.50	18.61	107.61	17.29	121.91	17.30	5.975ˆ**
Conscientiousness-NEO	113.68	23.40	112.93	20.14	114.14	25.24	0.373
Extraversion –EPQ	8.61	3.12	9.26	2.63	8.22	3.33	-2.445ˆ*
Neuroticism –EPQ	4.78	3.57	4.12	3.36	5.19	3.65	2.181ˆ*
Psychoticism-EPQ	3.00	2.46	3.69	2.56	2.59	2.30	-3.317ˆ*
Premeditation-UPPS	31.01	5.21	30.44	4.41	31.36	4.38	-1.505
Negative urgency-UPPS	27.00	3.12	26.48	5.22	27.33	5.19	-1.181
Sensation seeking-UPPS	31.59	7.58	34.75	7.24	29.66	7.14	-5.123ˆ**
Perseverance-UPPS	25.35	3.18	25.02	3.15	25.55	3.20	1.198
Positive urgency-UPPS	26.48	7.62	27.96	7.32	25.58	7.68	-2.284ˆ*
DAT-RA	23.95	6.58	25.51	6.00	22.99	6.76	-2.808ˆ**
SGG Panel 1 probability	0.48	0.25	0.44	0.26	0.51	0.24	-2.377ˆ*
SGG Panel 2 probability	0.43	0.20	0.42	0.20	0.44	0.21	0.787
SGG Panel 3 probability	0.42	0.20	0.37	0.21	0.45	0.20	-2.613ˆ**
SGG Panel 4 probability	0.40	0.21	0.36	0.20	0.43	0.21	-2.267ˆ*
SGG Panel 5 probability	0.54	0.28	0.52	0.32	0.55	0.26	-0.813
SGG Panel 6 probability	0.50	0.27	0.49	0.28	0.51	0.26	-0.524
SGG Panel 7 probability	0.48	0.25	0.46	0.28	0.49	0.23	-1.205
SGG Panel 8 probability	0.44	0.23	0.42	0.24	0.45	0.22	-0.962
HL Number of Safe Lotteries	6.79	1.97	6.39	2.27	7.03	1.73	-1.903ˆ+

*^+^p < 0.10; ^∗^p < 0.05; ^∗∗^p < 0.01.*

### Factor Analysis

When the factor analysis was performed, the first four factors presented eigenvalues greater than 1, and the parallel analysis suggested retaining four factors. The Barlett’s test for sphericity (χ^2^ = 1654, 563; df = 105, p<0.000) and the Kaiser-Meyer-Oklin (KMO = 0.729) indicated that the extraction method used was adequate to the data. **Table 2** shows the factor loadings of the personality scales in the factor solution. The factors corresponded to Unconscientious disinhibition, Neuroticism/negative emotionality, Extraversion/positive emotionality and Disagreeable disinhibition and accounted for 60% of the total variance.

**Table 2:** Principal axis factor analysis with varimax rotation of the personality scales.

	Factor 1	Factor 2	Factor 3	Factor 4 0
Conscientiousness NEO	**–0.86**	–0.28	0.01	–0.06
Premeditation UPPS	**–0.78**	0.07	–0.18	0.02
Perseverance UPPS	**–0.73**	0.04	0.08	0.02
Psychopathy secondary LSRP	**0.56**	**0.48**	–0.02	0.26
Psychoticism EPQ	**0.51**	0.10	0.16	**0.33**
Neuroticism NEO	0.07	**0.81**	**–0.34**	–0.07
Neuroticism EPQ	–0.03	**0.77**	–0.25	0.06
Negative urgency UPPS	0.10	**0.74**	0.15	0.20
Positive urgency UPPS	0.19	**0.43**	0.25	**0.34**
Extraversion NEO	–0.12	–0.16	**0.89**	–0.03
Extraversion EPQ	–0.01	–0.20	**0.77**	–0.01
Sensation seeking UPPS	0.19	0.06	**0.53**	0.27
Openness NEO	0.16	0.06	**0.46**	–0.25
Psychopathy primary LSRP	0.07	0.07	0.02	**0.92**
Agreeableness NEO	–0.09	–0.16	0.09	**–0.70**
Exp. Var.	24.26%	17.16%	10.48%	8.16%

*Bold, loadings higher than 0.30. Exp. Var., percentage of variance explained. Factor 1, unconscientious disinhibition; Factor 2, negative emotionality; Factor 3, positive emotionality; Factor 4, disagreeable disinhibition.*

### Mean Comparisons

**FIGURE 2:**
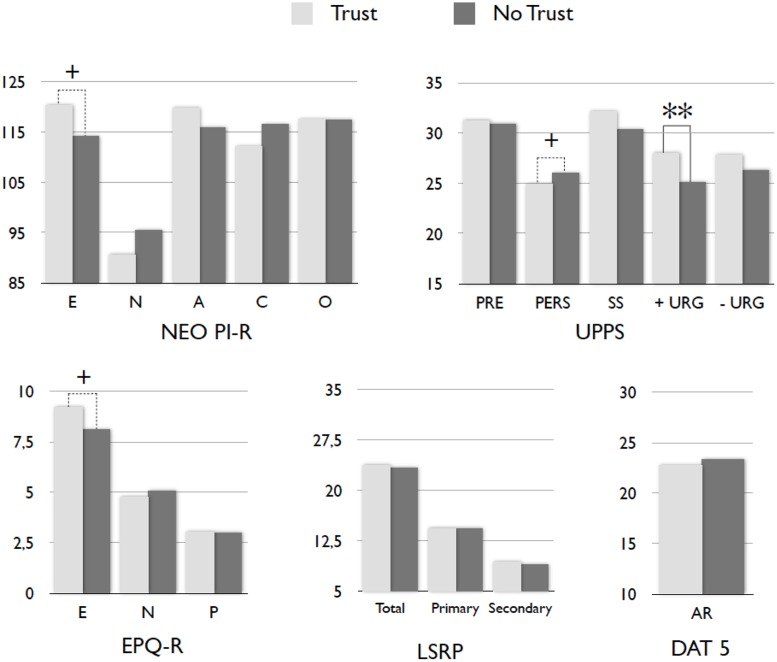
**Mean differences in personality scores between non-trusting** (*N* = 58) and trusting (*N* = 52) P1 players. E, extraversion; N, neuroticism; A, agreeableness; C, conscientiousness; O, openness; PRE, premeditation; PERS, perseverance; SS, sensation seeking; - URG, negative urgency; + URG, positive urgency; P, psychoticism; Total, psychopathy; Primary, primary psychopathy; Secondary, secondary psychopathy; AR, abstract reasoning. ^+^*p* < 0.10, ^∗∗^*p* < 0.01.

First, we split the sample of P1 players according to their strategy. Factor scores presented statistical differences between those participants who trust vs. those that do not trust in the *Extraversion/positive emotionality* factor (*t* = 2.117; *p* = 0.037). **Figure 2** shows that trusting and non-trusting P1 subjects exhibited similar means in all personality characteristics except in positive urgency, in which players who trust scored higher than non-trusting players. In addition, trustors also presented a non-significant tendency in the E dimension of both EPQ-R and NEO PI-R questionnaires (*p* = 0.06 and *p* = 0.10, respectively). When focusing on specific facets, trusting participants scored significantly higher in the *Warmth* facet of the E dimension than non-trusting participants (*t* = 2.020; *p* = 0.046) and showed a non-significant tendency in scoring lower on *Angry-hostility* facet of the N dimension (*t* = -1.820; *p* = 0.072).

**FIGURE 3:**
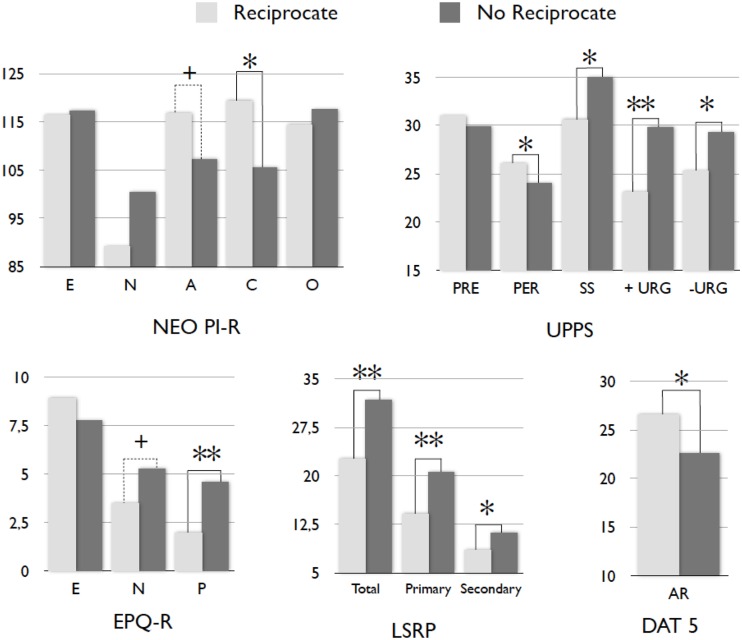
**Mean differences in personality scores between non-reciprocating (*N* = 33) and reciprocating (*N* = 19) P2 players.** E, extraversion; N, neuroticism; A, agreeableness; C, conscientiousness; O, openness; PRE, premeditation; PERS, perseverance; SS, sensation seeking; - URG, negative urgency; + URG, positive urgency; P, psychoticism; Total, psychopathy; Primary, primary psychopathy; Secondary, secondary psychopathy; AR, abstract reasoning. ^+^*p* < 0.10, ^∗^*p* < 0.05, ^∗∗^*p* < 0.01.

We split now the sample of active, deciding (*N* = 52) P2 players according to their strategy in the second stage of the game. Factor scores presented statistical differences between trustees that reciprocate *vs.* non-reciprocate in the *Disagreeable disinhibition* factor (*t* = -2.885; *p* = 0.006) and *Negative emotionality* factor (*t* = -2.449; *p* = 0.018), whereas *Unconscientious disinhibition* factor also presented a non-significant tendency (*t* = -1.911; *p* = 0.062). **Figure 3** depicts the mean differences in specific scales. Thus, it can be observed that trustees who display a reciprocal behavior have significantly lower levels in psychopathy-related traits than subjects who have opted for the individualistic reaction to their trusting counterpart. These differences are evident on the primary and secondary psychopathy and on P. Players who reciprocate also presented a non-significant tendency in A, mainly attributed to the significant mean differences found in the A facet of *Straightforwardness* (*t* = 2.611; *p* = 0.012). In addition, players who did not reciprocate presented higher scores on disinhibition-related traits, as positive and negative urgency, low persistence, sensation seeking, low C and the *Impulsivity* scale of N (*t* = 2.129; *p* = 0.038).

### Regression Analysis and Correlations

We present in **Table 3** the predictive power of the factors underlying the questionnaires on trust and reciprocity behaviors. Despite gender differences found in predictors, neither age nor gender associate to any dependent variable. Once controlled for these variables, neither cognitive ability nor risk aversion associate with trust, but higher AR predicted higher reciprocation. Regarding personality, the *Positive emotionality* factor that included E scales, predicted trust behavior, whereas *Disagreeable disinhibition* factor, which included primary psychopathy, P, positive urgency and low A scales, predicted non-reciprocation. In addition, Unconscientious disinhibition and Negative Emotionality factors presented a marginally non-significant association with no reciprocation behavior, probably reflecting the role of impulsivity on this behavior.

**Table 3:** Hierarchical Logistic Regression analysis with Trust and Reciprocate behavior as dependent variables.

		Trust (52 vs. 58)		Reciprocate (33 vs. 19)	
		*R*^2^ (Cox and Snell-Nagelkerke)	B	*R*^2^ (Cox and Snell-Nagelkerke)	B
Step 1	Gender (0 = male; 1 = female)	0.003–0.004	-0.232	0.003–0.004	-0.360
	Age		0.000		-0.086
Step 2	Fluid intelligence	0.005–0.007	-0.015	0.107–0.146^∗^	0.102^∗^
Step 3	Unconscientious disinhibition	0.056–0.075	-0.182	0.322–0.441^∗∗^	-0.738^+^
	Negative emotionality		-0.003		-0.693^+^
	Positive emotionality		0.485^∗^		0.276
	Disagreeable disinhibition		-0.096		-1.057^∗^
Step 4	Risk aversion HL	0.066–0.088	-0.022	0.337-0.462	0.038
	Risk aversion F1 SGG		-0.128		0.042
	Risk aversion F2 SGG		0.058		-0.432

Gender, age, fluid intelligence and personality factor scores as predictors (N in parenthesis). ^+^p < 0.10, *p < 0.05, **p < 0.01.

We look now at the results obtained from the physiological data. Interbeat intervals, measured one second after a screen is shown to P1 asking them to make a decision, significantly and negatively correlate with primary (Spearman, -0.338, *p* = 0.007) and total (Spearman, -0.314, *p* = 0.013) LSRP scores. Also, the amplitude of SCR corresponding to the same moment significantly correlates with primary (Spearman, 0.267, *p* = 0.015) and total (Spearman, 0.235, *p* = 0.033) LSRP scores. Both patterns indicate the relevance of the decision to trust in terms of attentional resources involved, and the emotions triggered in conjunction with the decision makers personality.

## Discussion

The present study addresses factors that can account for individual differences in behavior of participants in the TG. To this end, we selected a wide range of personality constructs that might be useful in explaining the heterogeneity observed.

In order to integrate the different personality characteristics assessed within the FFM framework, we performed an exploratory factor analysis. We found a four-factor structure virtually identical to the one described by Markon et al. (2005) and similar to the ones found in other studies with a wide variety of personality scales (e.g., Zuckerman et al., 1993; Ortet et al., 2002; Aluja et al., 2004; Ibáñez et al., 2010). According to the nomenclature in Markon et al. (2005), the four factors we obtained were labeled Positive Emotionality, Negative Emotionality, Disagreeable Disinhibition and Unconscientious Disinhibition. These factors are closely linked to the FFM of personality except for O, probably because this domain is not well represented in other personality models apart from the FFM (Markon et al., 2005).

Particularly relevant for the present research was the location of impulsivity and psychopathy scales within the FFM space. In reference to psychopathy, we found that subscales of the LSRP, although interrelated, loaded in two different factors: primary psychopathy characterized as manipulation, cheating, callousness and lack of remorse loaded in the Disagreeable Disinhibition factor, and would be mainly related to low A; while secondary psychopathy, characterized by impulsivity and deviant behavior, loaded in the Unconscientious Disinhibition factor and would be mainly related to low C, in line with previous findings (Miller et al., 2008). In relation to impulsivity, it constitutes a complex multifaceted construct of pervasive importance in psychology (Evenden, 1999). In an attempt to add clarity to the impulsivity concept, Whiteside and Lynam (2001) identified four distinct components of impulsivity (i.e., urgency, sensation seeking, perseverance, and deliberation) and located them within the FFM framework. Posterior studies subdivided urgency in two facets, negative urgency, and positive urgency (Cyders et al., 2007; Cyders and Smith, 2008). These facets were conceived as reflecting different ‘pathways’ to impulsive behavior. Accordingly, we found perseverance and deliberation to be closely linked to C, sensation seeking to E, and negative and positive urgency to N, although positive urgency would also be associated to low A and low C, in line with past research (Cyders and Smith, 2008).

With respect to the individual differences in the TG, first we deal with trusting behavior. Different approaches have been proposed to define and explain trust behavior (see Bauer, 2015). Recently Thielmann and Hilbig (2015b) have systematically reviewed the multiple basic processes underlying trusting behavior among strangers and its relationship to personality characteristics. They proposed that four main components would be relevant in the decision to trust: (a) attitudes toward risky prospects (i.e., risk aversion and loss aversion), (b) betrayal sensitivity, (c) trustworthiness expectations, and (d) sensitivity to reward. Importantly, individual differences in these processes would be casually linked to personality characteristics, so examining the relationship between personality and trust behavior would help in determining which of these mechanisms could be more relevant in the TG.

According to our results, the main mechanisms involved in trusting behavior in our experiment would be Reward sensitivity. Thielmann and Hilbig (2015b) suggested that some individuals might place attention on the potential reward inherent in a positive social interaction, so, individuals more sensible to reward, i.e., scoring high in Extraversion-related traits, should perceive social interactions as particularly rewarding *per se* and therefore be highly motivated to approach such interactions (Depue and Collins, 1999; Denissen and Penke, 2008). Accordingly, we found that *Extraversion/positive emotionality*, and specifically the facet of *warmth* associate to trust. People scoring high in warmth are friendly, easily forming close attachment to others (Costa and McCrae, 1999). In accordance to our results, some other studies have also found a similar role of E on trusting behavior (Swope et al., 2008; Ben-Ner and Halldorsson, 2010; Haring et al., 2013), suggesting that trustors’ investments have a component of facilitation of social relations by expecting a large gain from trust. This interpretation would be reaffirmed by the fact that we have also found an association of trust and positive urgency, the tendency to engage in rash action in response to high positive affect (Cyders and Smith, 2008), suggesting that part of this behavior is linked to a non-deliberative rash behavior in front of a perceived appetitive situation.

In contrast to our hypothesis, we have not found any association between A and trust. The hypothetical process underlying the relevance of A on trust would be the development trustworthiness expectations via social projection. To form an expectation about the other’s likely behavior, the trustor can consider different sources of information, as trust cues (i.e., reputation), prior trust experiences, or social projection (Thielmann and Hilbig, 2015b). Social projection implies that people would predict others cooperativeness by projecting their own cooperative preferences onto them (Krueger, 2013). In terms of the FFM, one’s cooperation and trustworthiness should be mainly covered by the A domain, so agreeable people would expect others to behave more cooperatively and reciprocate. Accordingly, Evans and Revelle (2008) and Becker et al. (2012) found a slight but significant effect of A on the amounts invested in the TG, and Müller and Schwieren (2012) confirmed the relevance of *trust* and *straightforwardness* for this behavior. However, in line of our results, other studies have not found association between A and investment behavior (Swope et al., 2008; Ben-Ner and Halldorsson, 2010; Haring et al., 2013). The fact that we and others have failed to find significant associations could be reflecting the difficulty in detecting modest effect sizes, as those described for the associations between A and investment behavior (Zhao and Smillie, 2015).

Our data also indicate the minor role on trust of the other two proposed mechanisms, betrayal sensitivity and attitudes toward risky prospects (Thielmann and Hilbig, 2015b). In terms of FFM, individual differences in these mechanisms would be linked to N, mainly the facet of *angry hostility* for betrayal sensitivity ((Maltby et al., 2008; Thielmann and Hilbig, 2015b) and the facet of *anxiety* for attitudes toward risk (Thielmann and Hilbig, 2015b). However, in line with previous findings (Evans and Revelle, 2008; Swope et al., 2008; Becker et al., 2012), no association between trust behavior and N-domain nor its facets are found. In addition, no association between trusting behavior and risk aversion measures have been found (Bohnet and Zeckhauser, 2004; Bohnet et al., 2008; Houser et al., 2010). These findings are important because they reinforce the idea that risk attitudes would not be successful in organizing trust behavior (Fehr, 2009). Thus, and to sum up, our data suggest that the more important mechanism underlying individual differences in the TG was sensitivity to reward. Attitudes toward risky prospects, betrayal sensitivity or trustworthiness expectations would exert a minor role, presenting low effect sizes that would be difficult to detect with the sample size used in the present research.

Once P1 has decided to cooperate (i.e., trust), P2 can exploit the other’s trust or can correspond with reciprocity. Reciprocity constitutes a key mechanism for explaining cooperative behavior among non-relatives, receiving strong attention from several disciplines, especially economics and evolutionary biology (Trivers, 1971; Fehr and Fischbacher, 2003; Falk and Fischbacher, 2006; Nowak, 2006; Tooby et al., 2006; Guala, 2012). Reciprocity could be understood as the tendency to respond “nicely” to nice actions (positive reciprocity) and “nastily” to nasty actions (negative reciprocity) when interacting with other players. Reciprocity can be beneficial for both parts (weak reciprocity), or even may involve a cost for responders (strong reciprocity). Cooperation usually emerges in repeated encounters within the same pair of individuals, helping each other (direct reciprocity). Nevertheless, cooperation is also extensively observed between strangers, probably because of an expected indirect gain (indirect reciprocity) like good reputation.

Conversely, a non-reciprocal subject may benefit from exploiting others’ trust. Exploitative behavior has received some attention recently, specifically from an evolutionary perspective (Mealey, 1995; Buss and Duntley, 2008; Lalumière et al., 2010; Glenn et al., 2011). According to this view, exploitation is a main class of strategies for acquiring reproductively relevant resources that consist in expropriating the resources of others through exploitation. This class of strategies ranges from mild, such as failing to reciprocate a minor favor in a social exchange, to extreme, such as coalitional warfare to expropriate all of an opposing group’s reproductively relevant assets (Buss and Duntley, 2008). The personality characteristic most strongly associated to exploitation would be low A and its extreme, psychopathy (Buss, 2009). From an evolutionary point of view, psychopathic behavior would constitute a successful alternative strategy at a low relative frequency in the population, whereby a small number of individuals take advantage of their more populous, cooperative counterparts by defecting in social interactions (Mealey, 1995; Lalumière et al., 2010; Glenn et al., 2011). Surprisingly, psychopathic traits had not been formerly explored in the TG so far.

As a result of our approach, we obtained the novel finding that those individuals that did not reciprocate were higher in *Disagreeable disinhibition*. Specifically, non-reciprocators scored higher in both primary and secondary Levenson psychopathy scales, P, and low C. Conversely, the decision to reciprocate in order to reward a kind action would depend on the A FFM dimension, and, specifically, on *straightforwardness*. Individuals scoring high in *straightforwardness* would be honest, sincere and ingenuous, whereas low scorers would be dishonest and would tend to manipulate others through flattery or deception (Costa and McCrae, 1999). Along this line, some studies have found that the most relevant personality domain for reciprocation is A (Ben-Ner and Halldorsson, 2010; Becker et al., 2012; Lönnqvist et al., 2012), especially its honesty aspects (Thielmann and Hilbig, 2015a). Thus, from an evolutionary personality perspective, reciprocal-exploitative behaviors would be located on a continuum of opposite strategies regarding behavior in cooperative situations, and the personality domain linked to this continuum would be the dimension of A.

In addition, our results also suggest that impulsivity would play a relevant role in trust and, especially in reciprocal behavior. To our knowledge, the present study is the first to systematically examine the role of this complex trait in the TG. We have found that a specific facet of impulsivity, positive urgency, is related to trusting behavior. Positive urgency refers to the tendency to engage in rash action as a response to high positive affect. This suggests that trusting behavior would be considered as a positive and potentially rewarding situation and that the decision to trust is partially guided by impulsive tendencies. In the same vein, reciprocal behavior also involves a non-reflexive component of the take-the-money-and-run type behavior, with individuals who are more sensitive to reward (sensation seeking), less perseverant, and score higher in urgency, both positive and negative, presenting rash responses of non-reciprocation. We think that these results, if replicated, could be theoretically relevant since they point to a the role of hot impulsive and non-reflexive mechanisms at the basis of trust (e.g., Murray et al., 2011) and reciprocity, in contrast to a more classical view of economic decisions associated with a more cold reflexive and calculative vision of human behavior.

The physiological results show that P1 participants scoring high in psychopathy exhibit increased EDA at the moment in which they are asked to decide whether to trust. At the same time, the P1 group show reduced evoked HR deceleration, indicating decreased attentional engagement during the decision-making process. Taken together, these two findings suggest that high psychopathy scorers perceive the decision-making task as less demanding compared to low-scorers, despite physiological changes signaling increased emotional arousal. No significant differences in EDA or HR variation arise between trusting vs. non-trusting or reciprocating vs. non-reciprocating participants.

According to the somatic marker hypothesis, decision-making is influenced by physiological signals that arise in bioregulatory processes, including those expressed as emotions (Damasio, 1996). Numerous studies have shown that emotional activation guides decision making in healthy subjects, while this effect is reduced in patients with orbitofrontal dysfunction (Bechara et al., 2000). Interestingly, psychopathic personality traits and antisocial behavior (clinical and sub-clinical) have been linked to orbitofrontal dysfunction (Dinn et al., under review). While previous research associated psychopathic behavior with reduced EDA (Lorber, 2004; Casey et al., 2013), our findings may indicate an alternative mechanism to promote antisocial behavior by suppressing the influence of somatic markers in decision making.

This study has several limitations. First, the magnitude of personality association with trust is modest and, therefore, some effects may not have been detected due to the relatively small sample size. Although the effects were greater in magnitude for reciprocal behavior, the reduced number of participants in the reciprocating and non-reciprocating groups led to a low statistical power in part of our analysis. Also in relation to the sample, it is important to highlight that our results are referred to non-clinical population, and therefore, the generalization to clinically relevant samples such as psychopaths should be made with caution. Another limitation, and a potentially source of discrepancies with other studies, is the discrete TG version used in present experiment, in contrast to the more usual continuous version used. Nevertheless, one strength of the present analysis is the inclusion not only of many personality domains, but also of specific traits relevant for particular behaviors (such as psychopathy for non-reciprocal behavior). However, and even though fluid intelligence has been used as a marker of general cognitive ability (Colom et al., 2007), other cognitive abilities have not been examined (McGrew, 2009). Thus, future research would benefit from including a larger number of participants, the use of clinical samples, and a broader selection of personality, economic and cognitive variables.

To conclude, although A and E are primarily dimensions of interpersonal behavior, E is related to the preferred quantity of social stimulation and A represents the characteristic quality of the interaction (Costa et al., 1991). Accordingly, the present study suggests that E could be relevant for initiating cooperation, whereas A could be relevant for maintaining it. That is, different personality domains would represent different strategies in the social domain, one based in the number of social contacts and the other in the cohesion of such contacts. With respect to the E domain, high E would favor a risky behavior that may increase the number of social partners. On the other hand, individuals scoring high in A would reward kind actions, even if this reward involves some cost for them. Conversely, low agreeable/high psychopathic and disinhibited/rash impulsive individuals would benefit from this situation, by taking the money and running!

## Ethics Statement

This study was carried out in accordance with the recommendations of the ethical committee at the Universitat Jaume I. The deputy chair of the LEE ethics committee, Dr. Eva Camacho led the process in this specific case. Participants gave written informed consent in accordance with the Declaration of Helsinki.

## Author Contributions

NG, GO, GS-G, and MI designed the general study. NG had the original idea of this specific paper. GS-G, SL-O, LM, and HV collected the data. MI, AG-G, IB-T, and LM performed the statistical analyses. PP designed, collected and analyzed the physiological data. MI, GS-G, and NG wrote the first manuscript draft. AG-G organized the database and coordinated the final version. All the authors contributed to and approved the final manuscript.

## Conflict of Interest Statement

The authors declare that the research was conducted in the absence of any commercial or financial relationships that could be construed as a potential conflict of interest. The reviewer PB and the handling Editor declared their shared affiliation, and the handling Editor states that the process nevertheless met the standards of a fair and objective review.

## Footnotes

1 Also called the ‘investment game.’

2 The data reported here are part of a larger study on personality traits and behavior in a series of games like PD, UG, Dictator and risky choice tasks. Payment was contingent on performance in one of all the economic games, chosen randomly at the end of the session, in order to avoid wealth accumulation effects and portfolio or hedging strategies. To avoid order effects, subjects were faced to the aforementioned contexts in randomized orders.

3 Software available upon request from A. Conde (alconvi@gmail.com) and J. V. Guinot (jose.guinot@gmail.com), JOOMALIA-Doing3D. The protocol used for the timing and communication between this software and the one used to measure the ECG is explained in detail in Perakakis et al. (2013).

4 Freely available at: https://github.com/mateusjoffily/EDA.

5 Attanasi et al. (2016) find no significant correlation between the two methods.

6 García-Gallego et al. (2012) provide detailed discussion on the multidimensionality of the test and its implications under expected utility and alternative theories of decision making under risk. Three different aspects of a subject’s risk attitude could be of interest here. First, whether a subject chooses safer choices. This would reflect a subject’s risk aversion. Second, the sensitivity of the subject’s choice to variations in t, measuring the incentive to take higher risks. Third, choice differences among gain (panels 1–4) and mixed-domain (panels 5–8) gambles, attributed to a subject’s loss aversion.

7 Whether the continuous version of the TG and its framing as a potentially reciprocal investment situation is more realistic, is a matter of the real-life example one has in mind. For example, there are situations in which a continuum of actions is not available and trust comes in the form of discrete events, like for example, signing first a contract or proposing marriage. In any case, the use of continuous vs. discrete versions could not be fully equivalent, and may led to somewhat different results. Thus, as observed by Schniter et al. (2016) when comparing this binary version of the TG with a continuous version, although investments are higher in the all-or-nothing game than in the continuous game, higher investments in the binary game do not lead to higher returns. This suggests that subjects perceive intentions not only by evaluating what others do but also by evaluating what others choose not to do.

8 See Markon et al. (2005) for a similar procedure.

9 For a similar rationale and procedure, see Ibáñez et al. (2010).
